# Impact of Rice and Potato Host Plants Is Higher on the Reproduction than Growth of Corn Strain Fall Armyworm, *Spodoptera frugiperda* (Lepidoptera: Noctuidae)

**DOI:** 10.3390/insects13030256

**Published:** 2022-03-04

**Authors:** Rajendra Acharya, Matabaro Joseph Malekera, Sanjeev Kumar Dhungana, Sushant Raj Sharma, Kyeong-Yeoll Lee

**Affiliations:** 1 Department of Applied Biosciences, College of Agriculture and Life Sciences, Kyungpook National University, Daegu 41566, Korea; racharya2048@gmail.com (R.A.); jmatabaro@live.com (M.J.M.); 2 Department of Southern Area Crop Science, Upland Crop Breeding Research Division, National Institute of Crop Science, Rural Development Administration, Miryang 50424, Korea; sanjeev@korea.kr; 3 CIMMYT International, South Asia Regional Office, Lalitpur 44700, Nepal; gpsharma019@gmail.com; 4 Institute of Plant Medicine, Kyungpook National University, Daegu 41566, Korea; 5 Graduate School of Plant Protection and Quarantine, Kyungpook National University, Daegu 41566, Korea

**Keywords:** *Spodoptera frugiperda*, life table, corn strain, survival, reproduction

## Abstract

**Simple Summary:**

Since 2016, the fall armyworm (FAW), an invasive pest native to tropical and subtropical regions of the Americas, has invaded Africa and further spread into Asian countries. FAW is a polyphagous species, although the invaded strain mostly damages corn rather than any other host plants. Studies on the biology of corn strain FAW reared on three different host plants: corn, rice, and potato, using the age-stage, two-sex life table, showed that growth, development, survival, and reproduction rate of the corn strain FAW were differentially affected by rice and potato host plants. The reproduction rate was highly affected than other parameters such as growth, development, survival rates. Our results provide important information for the understanding of the population dynamics of FAW and an appropriate management strategy in the newly FAW-invaded agricultural ecosystems.

**Abstract:**

The fall armyworm (FAW), *Spodoptera frugiperda*, is an invasive pest species that has recently increased its range in most African and Asian countries, causing significant losses to crop yields, especially corn. To develop effective management strategies, it is particularly important to study the biology of FAW in various crops. Here, we utilized the age-stage, two-sex life table to examine the development, survival, and reproduction rate of the corn strain FAW on three different host plants: corn, rice, and potato. The corn strain FAW successfully completed its life cycle in rice and potato, as well as corn plants. However, the growth, developmental time, survival, and reproduction rate differed among the three host plants. The preadult survival rates in corn, rice, and potato were 92%, 81%, and 77%, respectively. Similarly, mean generation time was significantly shorter in corn (35 days), followed by rice (41 days) and potato (42 days), indicating more generations in corn. Interestingly, the net reproduction rate varied greatly among the three host plants. In corn-fed FAW, the net reproduction rate was 472 offspring per individual, whereas, in rice and potato crops, the rates were only 213 and 86 offspring per individual, respectively. Our results suggest that alternative host plants, such as potato and rice, have more effect on reproduction than the growth of corn strain FAW. These results may be useful in predicting the population dynamics of FAW and understanding the potential damage to crops, thus contributing to an appropriate management strategy in the newly FAW-invaded agricultural ecosystems.

## 1. Introduction

The fall armyworm (FAW), *Spodoptera frugiperda* (Smith 1797) (Lepidoptera: Noctuidae), is native to tropical and subtropical regions of the Americas. However, in 2016, it invaded Africa and rapidly spread through most African countries and later in Asian countries [1]. FAW invaded India in 2018 [2] and spread out across most Asia–Pacific countries, including Korea, Japan, and Australia, in early 2019 [3,4,5,6]. FAW is a polyphagous species that has been found in more than 350 host plants [7,8] and has been reported to have caused up to 73% grain yield losses in corn cultivation in Central America [9]. FAW mainly consists of two genetically different strains, known as corn strain (C-strain) and rice strain (R-strain). The C-strain is found more commonly on corn, cotton, and sorghum, whereas R-strain is found on rice and grasses [7,10,11].

As FAW is a serious invasive pest, the development of effective management strategies in new habitats is necessary. To achieve this goal, it is useful to first establish a basic knowledge of the biological and ecological parameters of this pest. In our previous study, all FAW samples collected in Asia were of the corn strain [3]. Corn and rice are staple food crops, and potato is one of the major vegetable crops in Asian countries. In tropical and subtropical areas of Asia, farmers rotate the cultivation of corn and rice in the summer with a crop of potatoes in the winter. Therefore, FAW populations can survive in these fields and cause damage yearlong. Moreover, both strains can cause damage to each type of host plant [7]. Juárez et al. [12] reported that both strains can infest both categories of host crops, and an undefined relationship between the hosts and FAW strains has been reported. Surprisingly, Zhao et al. [13] and Sun et al. [14] found that potato fields in China were damaged by FAW.

The various species of host plants may significantly affect the growth, developmental time, survival rate, and reproduction rate of herbivorous insects [15]. In the design and implementation of pest management strategies, consideration should be given to the influence of host species that may inhibit or promote insect growth. Accordingly, an investigation of the influence of these host plants on the fitness of FAW is warranted.

Life tables are considered the key tools for understanding insect population ecology and designing effective pest management techniques. This approach provides comprehensive information on the insect population dynamics and their fitness under different conditions by incorporating all life-history parameters, including the growth, development, survival, and reproduction of both sexes. Furthermore, it is important to understand the life table of insect pests because the growth rate, developmental time, survival, and reproduction rate determine the population dynamics, which is of utmost importance when developing strategies for pest management [16]. Traditional female-based age-specific life tables [17,18,19,20] ignore the stage differentiation and contribution of males in a population, leading to an improper description of the population characteristics [21]. In contrast, the age-stage, two-sex life table [22] incorporates the contributions of both sexes and illustrates the stage differentiations.

In this study, we analyzed the life table data of FAW reared on corn, rice, and potato and evaluated the population fitness using the age-stage, two-sex life table approach described by Chi [23]. This is the first such study on the comparative investigation of life tables of corn strain FAW fed on corn, rice, and potato, using this approach. Our research aimed to provide detailed comparable information about the FAW population growth and possible damage to corn, rice, and potato crops, with the goal of providing helpful information for the implementation of effective FAW management strategies in the newly FAW-invaded agricultural ecosystems.

## 2. Materials and Methods

### 2.1. Insect Culture

FAW larvae were collected from a cornfield in Gyeongsan, Gyeongbuk Province, Korea, in August 2019 and were identified as the corn strain by utilizing *triosephosphate isomerase* (*Tpi*) gene (GenBank Accession #MT894238) and mitochondrial *cytochrome oxidase subunit I* (*COI*) gene (GenBank Accession #MT103342) sequence analysis [3]. The insect colony was maintained under controlled conditions of 25 ± 1 °C, 60 ± 5% relative humidity (RH), and 14/10 h light/dark cycles. The larvae were fed on an artificial diet (Product number F9772; Frontier Scientific Services, Newark, DE, USA) prepared with 3.8 g agar, 28.80 g dry mix applicable to lepidopteran insects, and 200 mL distilled water. Adult moths were fed on 20% sugar solution [24,25].

### 2.2. Host Plants

Corn cv. Dehakxil, rice cv. Hyunmi, and potato cv. Sumi were grown in plastic pots filled with nursery media (Punong, Gyeongju, Korea) containing 68% coco peat, 7% perlite, 14.729% peat moss, 3% vermiculite, 7% zeolite, 0.243% fertilizer, 0.024% pH regulator, and 0.004% moisture. The pots were kept in a plant growth chamber adjusted to 25 ± 2 °C, 60 ± 5% RH, and 16/8 h light/dark cycles. The FAW larvae were provided with the following food: leaves of corn at the V6 stage, potato plants at 30–40 cm height, and rice at the tillering stage.

### 2.3. Measurement of Larval and Pupal Weight

The first instar larvae were allowed to feed on the leaves of each crop plant separately. The body weight of the 4th to 6th instar larvae (*n* = 25) and pupae (*n* = 20) was measured at 24 h after molting from the previous developmental stage.

### 2.4. Life Table Study

The development, survival, and reproduction of FAW were evaluated in the different host plant treatments: corn, rice, and potato. Two egg clusters, each containing ~200 eggs, were transferred into the insect breeding dish lined with moist filter paper. Individual neonates were transferred into plastic cups with small holes for aeration. A total of 78, 72, and 70 neonates were used for corn, rice, and potato food, respectively. To avoid microbial contamination and ensure enough food for the larvae, fresh leaves washed with tap water were provided daily. Individual larvae were regularly observed, and molting and survivorship were noted. The developmental stages of larvae were identified based on the removal of the head capsule. After the feeding of the larvae was completed, the prepupae were provided with the autoclaved nursery media (Punong, Gyeongju, Korea) for pupation. Each pupa was sexed, weighed, and placed individually in a plastic cup containing autoclaved nursery media. Newly emerged adults from the same plants were paired and were released in an acre cage, followed by regular counting of the number of eggs.

### 2.5. Life Table Data Analysis

Raw data of the development, survival, and reproduction of FAW were analyzed using the TWO-SEX-MSChart program [26], as described by Chi [23]. The survival rate (*s_xj_*) (*x* = age, *j* = stage) and fecundity (*f_xj_*) were analyzed as suggested by Chi and Liu [22].

The bootstrap method, with 100,000 re-samplings, was used to estimate the standard error of all life table parameters [27,28]. Paired bootstrap was used to compare the mean values among the host plants. All the charts were created using SigmaPlot v.12.5 software.

## 3. Results

### 3.1. Body Weights of Larvae and Pupae of Fall Armyworm

**Figure 1:**
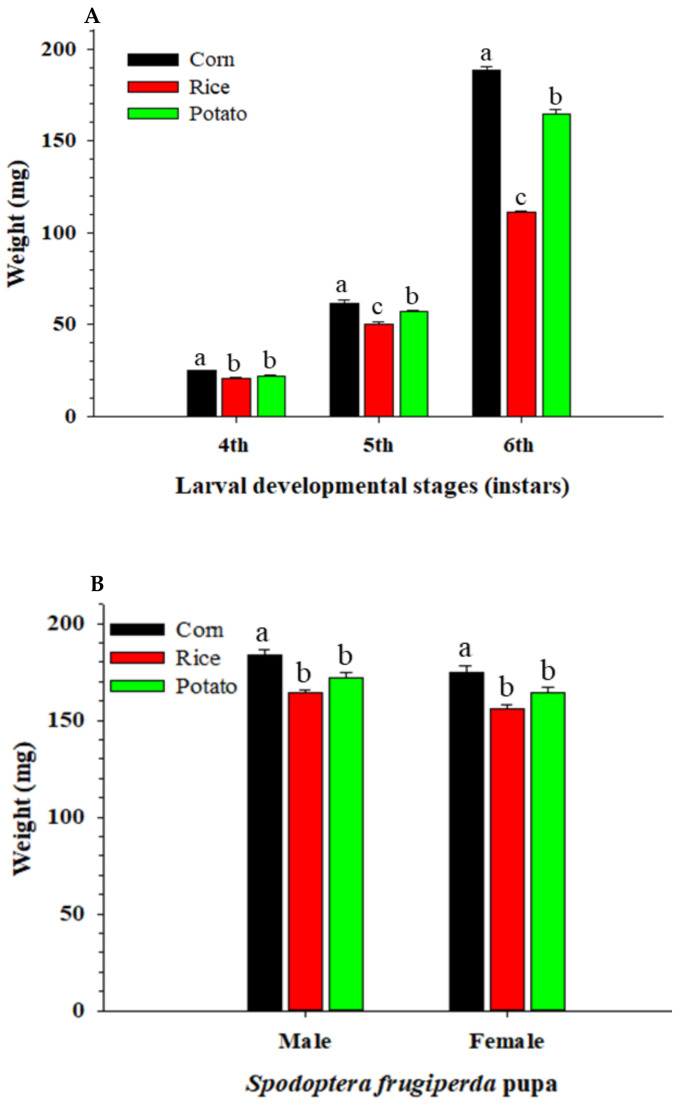
Larval (**A**) and pupal (**B**) weight of *Spodoptera frugiperda* fed on corn, rice, and potato. Different letters above bars within the same instar stages or sexes denote a significant difference.

From the 4th to 6th instar larval stages, the body weights were significantly (*p* < 0.0001) higher in the corn treatment group than in the potato and rice groups (Figure 1A). In particular, the difference in body weight among the three plants was greater in the older larval stages. In terms of body weight of both male and female pupae, the pupal biomass was significantly (*p* < 0.0001) higher in corn than in potato and rice, but there was no significant difference between the potato and rice groups (Figure 1B).

### 3.2. Development, Survivorship, and Reproduction of Fall Armyworm

The developmental periods of all immature stages, adult longevity, and female fecundity of FAW feed on corn, potato, and rice are summarized in Table 1. Eggs were collected from the colony, and eggs hatched on the same day were used for the experiments. The duration of egg hatching in all three treatment groups was 3 days. Among the three host plant species, the developmental period of preadult stages of FAW was significantly shorter (*p* = 0.0001) in the corn plant group (29.75 days), followed by rice (35.19 days) and potato (36.87 days). The survival rate of preadult stages was higher (*p* < 0.0001) in the corn group (91%), followed by rice (81%) and potato (77%) groups. The adult longevity for both sexes was significantly higher (*p* < 0.0001) in larvae-fed corn than those reared on rice and potato. The mean fecundity was significantly higher (*p* < 0.0001) in FAW reared on corn (955.62 offspring/female than FAW reared on rice (590.77 offspring/female) and potato (231.54 offspring/female). The adult preoviposition period (APOP) and total preoviposition period (TPOP) were significantly different among FAW reared on corn, rice, and potato. A significantly (*p* < 0.0001) longer oviposition duration was observed for corn (7.68 days), followed by rice (5.83 days) and potato (3.23 days).

### 3.3. Population Parameters on Corn, Potato, and Rice Plants

The population parameters of FAW on corn, potato, and rice plants are presented in Table 2. The values of the intrinsic rate of increase (*r*) (0.17 per day), finite rate of increase (*λ*) (1.19 per day), net reproductive rate (*R_0_*) (472.28 offspring per individual) were significantly (*p* < 0.0001) higher on corn than on rice and potato. The mean generation time (*T*) was significantly (*p* < 0.0001) lower on corn plants (35.22 days) compared to rice (41.19 days) and potato (41.89 days).

### 3.4. Life Table Study of Fall Armyworm

**Figure 2:**
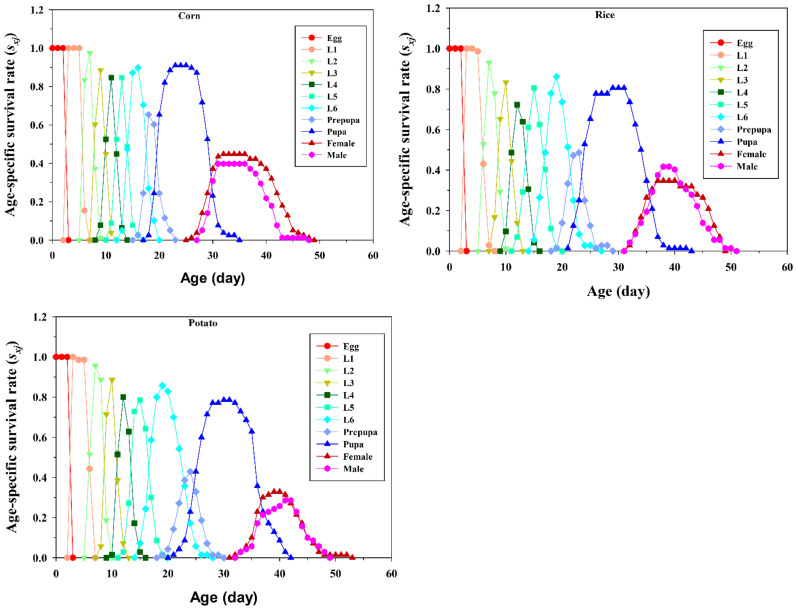
Age-stage-specific survival rate (*s_xj_*) of *Spodoptera frugiperda* fed on corn, rice, and potato. Curves of *s_xj_* depict the probability that a newborn will survive to age *x* and stage *j* (where L1–L6 are the larval instars from first to sixth). The various developmental rates among individuals overlap between different stages at the time of developmental periods.

The age-stage-specific survival rate (*s_xj_*) denotes the probability that a newly hatched FAW egg will survive to age *x* and stage *j* on corn, potato, and rice plants (Figure 2). Due to the variable developmental rates among the individuals, overlaps of the stage-specific survivorship curves were observed in all three plant groups. The probability that newly hatched FAW eggs will survive to the adult stage was significantly (*p* < 0.0001) higher in the corn group (91.03%) compared to the potato (77.14%) and rice (80.56%) groups.

**Figure 3:**
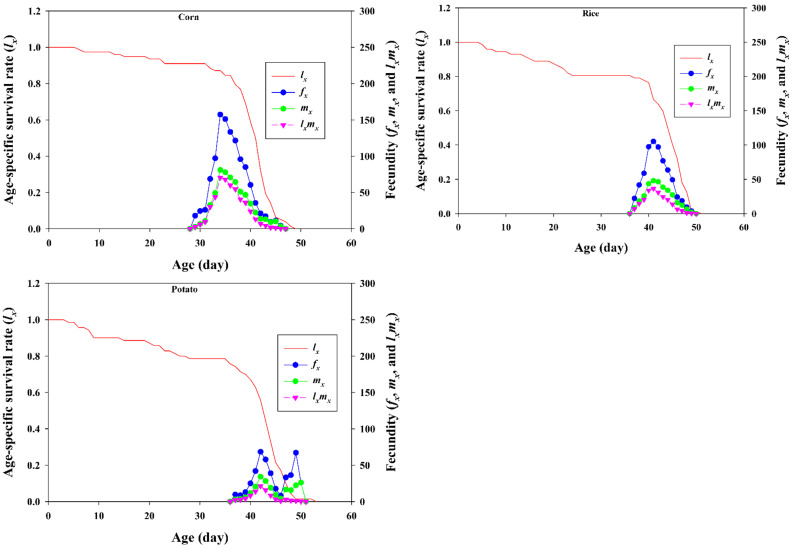
Age-specific survival rate (*l_x_*), female age-specific fecundity (*f_x_*), age-specific fecundity (*m_x_*), and age-specific maternity (*l_x_m_x_*) of *Spodoptera frugiperda* fed on corn, rice, and potato.

We then compared the age-specific survival rate (*l_x_*), the age-specific fecundity (*m_x_*), the female age-stage-specific fecundity (*f_x_*), and the age-specific net maternity value (*l_x_m_x_*) of FAW reared on corn, potato, and rice (Figure 3). The *l_x_* curve slightly decreased and remained at 91% up to the first 31 days in the corn group, whereas the *l_x_* curve rapidly decreased to below 90% within 15 days in the potato group and 16 days on rice. The *m_x_* curve denotes the age-specific fecundity of the cohort at the age *x* and the *f_x_* curve denotes the number of fertilized eggs laid by a female at age *x*. Both curves showed similar patterns for all three crops. In corn, reproduction began at age 28 days, and the highest peak was observed at the age of 33 days, with 80.99 eggs. In contrast, reproduction began at age 36 days in both the potato and rice groups, and the highest peak was observed at age 41 and 40 days, with mean fecundity of 34.20 and 47.87 eggs, respectively. In addition, the age-specific maternity (*l_x_m_x_*) of corn-fed FAW was higher than in the rice- and potato-fed FAW.

**Figure 4:**
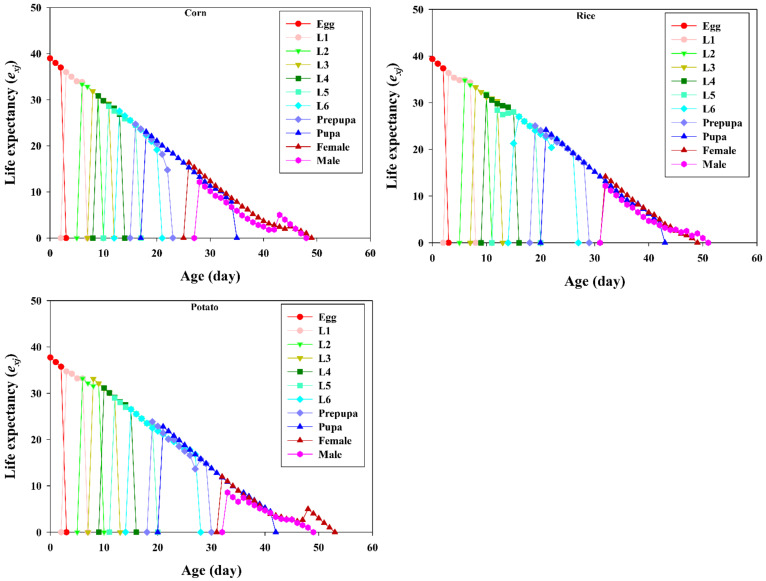
Age-stage-specific life expectancies (*e_xj_*) of *Spodoptera frugiperda* fed on corn, rice, and potato. L1–L6 are the larval instars from first to sixth.

The life expectancy (*e_xj_*) describes the length of time that an individual of age *x* and stage *j* is expected to survive (Figure 4). The life expectancy values of newly laid eggs of FAW that were reared on corn, potato, and rice were 38.99, 37.33, and 39.36 respectively. The life expectancy of newly emerged individuals was statistically identical to the mean longevity of all individuals used in this study.

**Figure 5:**
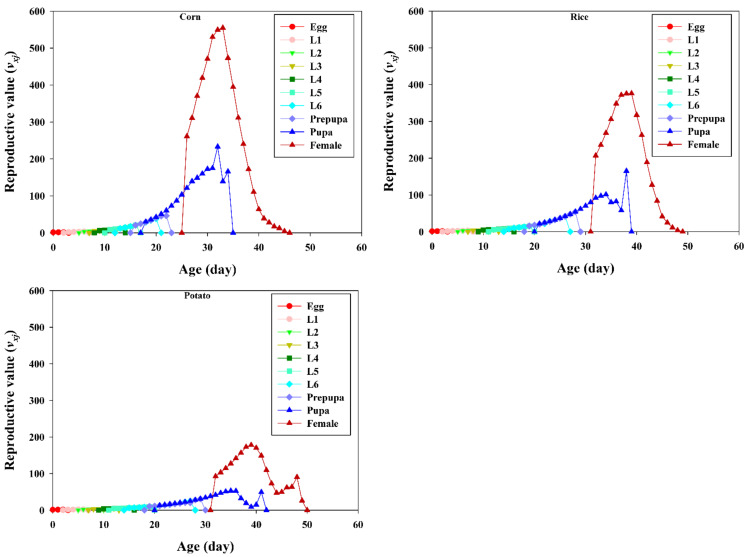
Age-stage-specific reproductive values (*v_xj_*) of *Spodoptera frugiperda* fed on corn, potato, and rice. L1–L6 are the larval instars from first to sixth.

The age-stage-specific reproductive value (*v_xj_*) describes the contribution of an individual of age *x* and stage *j* to the future population (Figure 5). The reproductive value increases with successive developmental stages; the highest reproductive peak value was observed at 33, 39, and 39 days, with 554.71, 177.9, and 376.14 eggs counted in the corn, potato, and rice groups, respectively.

## 4. Discussion

The biological characteristics such as growth, development, survival, and reproduction of phytophagous insects are greatly affected by the host plant species [29]. Each host plant species consists of various nutritional compounds and also contains various secondary metabolic compounds that have different defensive mechanisms such as tolerance, antibiosis, and antixenosis [30,31]. The results of our study of FAW at different life stages illustrate this relationship; although the population of corn strain FAW completed its life cycle in all three crops (corn, potato, and rice), the biological characteristics varied significantly among the three host plants.

The life parameter statistics, *R_0_*, *r*, *λ*, and *T*, indicate the capacity of growth for a given population in a specific environment [32]. These parameters often vary with host plant species and local environmental conditions [33,34,35]. The longer developmental time, lower survival rate, lower oviposition period, and lower fecundity rate of FAW reared on potato and rice resulted in lower *R_0_*, *λ*, and *r* values and higher *T* values. These trends might be due to the nutritional differences in the host plants [36]. In the present study, the shorter preadult duration, longer adult longevity, and higher fecundity of FAW reared on corn suggest that corn is a highly susceptible host plant compared to rice and potato. Additionally, the longer oviposition duration, longer TPOP, shorter APOP, shorter mean generation time, and higher population parameters (*R_0_*, *λ*, and *r*) are additional evidence that corn is a more suitable and susceptible host than are rice and potato. Interestingly, the net reproductive rate (*R_0_*) varied greatly among the three host plants. The number of offspring per individual in the group fed corn was 472, whereas the corresponding number in the rice- or potato-fed populations was only 213 and 86 respectively. These values were 2.22 and 5.49 times lower in the rice and potato treatments than in corn-fed FAW. Therefore, the reproduction rate of corn strain FAW was significantly reduced when they fed rice or potato. Similarly, Guo et al. [37] also reported that corn-fed FAW populations showed a greater reproductive rate than potato-fed populations. This result suggests that alternative host plants—such as potato and rice—had negative effects on the reproduction of corn strain FAW. However, a more focused study on the effect of alternative host plants on the regulation of FAW reproductive hormones is required to ascertain the mechanism behind this effect.

The FAW group reared on corn had 10.0% and 14.0% higher preadult survival rates than the rice and potato groups. In particular, a lower survival rate of larvae on potato and rice plants was observed at the younger larval stages (first to third instar larvae). Similar results were reported by Guo et al. [37]. The authors observed that first- and second-instar larvae fed on potato and tobacco leaves had a low survival rate compared with those fed on corn leaves. This may be due to the differences in host plant suitability in the younger stages of larvae [38]. The preadult duration of FAW was 29.75, 35.19, and 36.87 days in the corn, rice, and potato groups, respectively. In agreement with the current study, Wang et al. [32] also reported a shorter preadult duration of FAW on corn (24.67 days) compared to tomato (38.06 days), Chinese cabbage (37.33 days), and wheat (25.18 days). Similarly, Silva-Brandão et al. [39] and Orcucci et al. [40] also reported that corn strain FAW performed better growth and development with higher survival rates on corn compared to rice. These differences among host plants can be caused by various factors, including intrinsic genetic characteristics, as well as extrinsic features of food sources. For example, the reduced performance found in rice and potato might be associated with poor nutrition and/or the presence of some insect-inhibiting compounds, such as proteinase inhibitors in potato [41,42] and schaftosides in rice [43].

## 5. Conclusions

In conclusion, this is the first comparison of life table parameters of corn strain FAW reared on corn, rice, and potato, using the age-stage two-sex approach. Our results showed that the corn strain FAW can complete their life cycle in all three host plants but had a shorter preadult duration, higher survival rate, and higher fecundity in corn compared to rice and potato. The combined effect of all these parameters resulted in higher *R_0_*, *λ*, and *r* in FAW reared on corn, which explains the strong adaptability of this pest in corn compared to rice and potato. The reproduction rate was more affected than growth, development, and survival rates. However, our results suggest that rice and potato crop plants may serve as alternative hosts in the fields because corn strain FAW can successfully complete their life cycle by feeding on rice and potato plants. Therefore, it is important to monitor the population dynamics and potential crop damage of corn strain FAW in alternative host plants. Our study provides important information in predicting the population dynamics of FAW and understanding the potential damage to crops, thus useful for the development of an appropriate management strategy in the newly FAW-invaded agricultural ecosystems.

## Figures and Tables

**Table 1:** Development duration (days), total preadult duration, adult longevity, adult preoviposition period, total preoviposition period, reproductive days, and fecundity of *Spodoptera frugiperda* fed on corn, potato, and rice.

Stage	Corn		Rice		Potato	
	*n*	(Mean ± SE)	*n*	(Mean ± SE)	*n*	(Mean ± SE)
Egg		3 ± 0.00 a		3 ± 0.00 a		3 ± 0.00 a
First instar	78	3.16 ± 0.04 a	72	3.48 ± 0.07 b	70	3.46 ± 0.06 b
Second instar	77	2.24 ± 0.05 a	69	2.66 ± 0.06 b	76	2.68 ± 0.06 b
Third instar	76	2.03 ± 0.06 a	68	2.37 ± 0.06 b	63	2.35 ± 0.06 b
Fourth instar	76	2.01 ± 0.02 a	67	2.46 ± 0.06 b	63	2.40 ± 0.07 b
Fifth instar	76	2.11 ± 0.04 a	67	3.18 ± 0.09 b	63	3.19 ± 0.08 b
Sixth instar	74	3.59 ± 0.08 a	65	4.53 ± 0.13 b	62	6.00 ± 0.14 c
Prepupa	74	2.04 ± 0.02 a	60	2.28 ± 0.06 b	59	2.27 ± 0.06 b
Pupa	72	9.58 ± 0.12 a	58	11.03 ± 0.12 b	55	11.57 ± 0.16 c
Preadult	72	29.75 ± 0.19 a	58	35.19 ± 0.27 b	55	36.87 ± 0.32 c
Preadult survival rate	72	0.91 ± 0.03 c	58	0.81 ± 0.05 b	55	0.77 ± 0.05 a
Adult longevity						
Female	37	12.86 ± 0.49 b	26	11.31 ± 0.62 b	26	8.04 ± 0.49 a
Male	34	10.17 ± 0.46 c	32	8.77 ± 0.41 b	28	6.18 ± 0.44 a
Mean fecundity	37	955.62 ± 58.72 c	26	590.77 ± 46.30 b	26	231.54 ± 28.48 a
APOP	37	2.92 ± 0.10 a	26	4.46 ± 0.16 c	26	4.05 ± 0.10 b
TPOP	37	32.41 ± 0.31 a	26	38.96 ± 0.37 b	26	40.32 ± 0.43 c
Oviposition days	37	7.68 ± 0.388 c	26	5.83 ± 0.28 b	26	3.23 ± 0.24 a

Prepupa denotes the stage from the last larval stage that is often quiescent to before the ecdysis to pupa. Preadult is the duration from the egg to adult emergence. Values are shown as mean ± standard error (SE). The values followed by different letters within the same rows indicate a significant difference.

**Table 2:** Population parameter of *Spodoptera frugiperda* fed on corn, potato, or rice plants.

Parameter	Corn	Rice	Potato
Cohort size (n)	78	72	70
Intrinsic rate of increase (*r*) (d^−1^)	0.17 ± 0.01 b	0.13 ± 0.01 a	0.11 ± 0.01 a
Finite rate of increase (*λ*) (d^−1^)	1.19 ± 0.01 b	1.14 ± 0.01 a	1.11 ± 0.01 a
Net reproductive rate (*R_0_*) (offspring/individual)	472.28 ± 62.89 c	213.33 ± 37.28 b	86 ± 16.98 a
Mean generation time (*T*) (d)	35.22 ± 0.30 a	41.19 ± 0.38 b	41.89 ± 0.48 b

Values are shown as mean ± standard error (SE). The values followed by different letters within the same rows indicate a significant difference.

## Data Availability

The data presented in this study are available on request from the corresponding author.
